# Enhanced electronic properties in mesoporous TiO_2_ via lithium doping for high-efficiency perovskite solar cells

**DOI:** 10.1038/ncomms10379

**Published:** 2016-01-13

**Authors:** Fabrizio Giordano, Antonio Abate, Juan Pablo Correa Baena, Michael Saliba, Taisuke Matsui, Sang Hyuk Im, Shaik M. Zakeeruddin, Mohammad Khaja Nazeeruddin, Anders Hagfeldt, Michael Graetzel

**Affiliations:** 1 Laboratory of Photonics and Interfaces, Institute of Chemical Sciences and Engineering, School of Basic Sciences, Ecole Polytechnique Fédérale de Lausanne (EPFL), Lausanne CH-1015, Switzerland; 2 Laboratoire des sciences photomoléculaires, Institute of Chemical Sciences and Engineering, School of Basic Sciences, Ecole Polytechnique Fédérale de Lausanne (EPFL), Lausanne CH-1015, Switzerland; 3 Group for Molecular Engineering of Functional Materials, Institute of Chemical Sciences and Engineering, School of Basic Sciences, Ecole Polytechnique Fédérale de Lausanne (EPFL), Lausanne CH-1015, Switzerland; 4 Advanced Research Division, Materials Research Laboratory, Panasonic Corporation, 1006, Kadoma, Kadoma City, Osaka 571-8501, Japan; 5 Department of Chemical Engineering, Kyung Hee University, 1732 Deogyeong-daero, Giheung-gu, Yongin-si, Gyeonggi-do 446-701, Republic of Korea

## Abstract

Perovskite solar cells are one of the most promising photovoltaic technologies with their extraordinary progress in efficiency and the simple processes required to produce them. However, the frequent presence of a pronounced hysteresis in the current voltage characteristic of these devices arises concerns on the intrinsic stability of organo-metal halides, challenging the reliability of technology itself. Here, we show that n-doping of mesoporous TiO_2_ is accomplished by facile post treatment of the films with lithium salts. We demonstrate that the Li-doped TiO_2_ electrodes exhibit superior electronic properties, by reducing electronic trap states enabling faster electron transport. Perovskite solar cells prepared using the Li-doped films as scaffold to host the CH_3_NH_3_PbI_3_ light harvester produce substantially higher performances compared with undoped electrodes, improving the power conversion efficiency from 17 to over 19% with negligible hysteretic behaviour (lower than 0.3%).

## Abstract (web summary)

Perovskite solar cells are still plagued by hysteresis, despite the good charge transport properties of the perovskite counterpart. Here, the authors dope the mesoporous TiO_2_ scaffold with lithium to improve the transport properties of this other important component of solar cells, and reduce the hysteresis.

## Graphical abstract

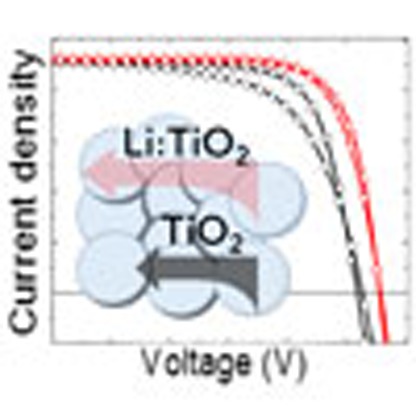

Perovskite-based solar cells (PSCs) have made impressive strides in just a few years with maximum power conversion efficiencies (PCEs) jumping from 3.8% (ref. 1) in 2009 to 20.1% (ref. 2) in 2015. Even though further improvements are still expected^3^, such rapid progress is unprecedented for any photovoltaic (PV) material. For instance, silicon, GaAs, CIGS and CdTe required decades to fully realize their potential as solar cells^4^.

Perovskites comprise a large family of crystalline materials, where the most commonly used for solar cells have an ABX_3_ chemical composition containing an organic cation A, such as methylammonium (MA) or formamidinium (FA)^5^,^6^, a divalent metal B, such as Pb or Sn^7^,^8^, and a halide X, such as Br or I. These organic–inorganic perovskites can be processed by a large number of techniques ranging from spin coating^5^, dip coating^9^, two-step interdiffusion^10^, chemical vapour deposition^11^, spray pyrolysis^12^, atomic layer deposition^13^, ink-jet printing^14^, to thermal evaporation^15^,^16^. The PV performances have been attributed to their outstanding optoelectronic properties such as remarkably high absorption over the visible spectrum^7^, charge carrier diffusion lengths in the micrometre-range^17^,^18^,^19^ implying a sharp optical band edge, and a tuneable band gap from 1.1 to 2.3 eV by interchanging the above cations^2^,^20^, metals^21^,^22^ and/or halides^23^.

Recently, Jeon *et al*. achieved one of the highest certified PCEs of 17.9% by using the mixed halide and cation formulation, (FAPbI_3_)_0.85_(MAPbBr_3_)_0.15_ (ref. 24). Their record solar cell architecture contains a mesoporous TiO_2_ layer, which is infiltrated by a liquid perovskite precursor solution forming the solid perovskite film after subsequent annealing. Mesoporous TiO_2_ has been widely used for high-surface-area electrodes, in optoelectronic applications^25^ and, in particular, in dye-sensitized solar cells (DSSCs)^26^, where they have been demonstrated to collect and transport electrons photoinjected from a surface-adsorbed sensitizer. One strategy to improve DSSCs was to enhance the electron transport of the mesoporous TiO_2_ making use of substiutional dopants^27^,^28^,^29^,^30^,^31^,^32^,^33^,^34^,^35^,^36^,^37^. Also, lithium intercalation has been employed to lower the conduction band edge of TiO_2_ facilitating electron injection and transport in the mesoporous TiO_2_ (refs 38, 39, 40, 41). By analogy, it may be expected that the introduction of n-dopants would also enhance the performance of PSCs. However, so far very few studies have examined the doping effect on the electron transport within the mesoporous TiO_2_ scaffold employed as PCSs^42^. This may be attributed to the perovskites already being excellent charge transporting materials inherently suited for electron conduction in high-efficiency solar cells^17^. This makes it challenging to investigate the effects of enhanced charge transport in mesoporous TiO_2_ as this improvement would only have a discernible impact for embodiments when the perovskite is operating at its limit.

Therefore, at first glance, it may be assumed that highly efficient PSCs could be achieved by depositing a perovskite film directly on a thin TiO_2_ compact layer, which would effectively work as an electron-selective contact^15^. However, flat junction PSCs prepared on a compact TiO_2_ suffer from relatively poor charge collection efficiency under steady-state forward voltage bias^43^. Although Wojciechowski *et al*. have shown significant improvement of this architecture by modifying the TiO_2_-perovskite flat heterojunction with fullerene derivatives^44^, the steady-state PCE of TiO_2_-based flat PSCs is still substantially lower than those based on mesoporous TiO_2_. The planar PSC architecutres are also plagued by severe hysteresis in the *J*–*V* curve rendering it very difficult to determine their solar to electric PCEs. For this reason, none of the TiO_2_-based flat PSC architectures have been certified so far. Recently, Guillén *et al*. demonstrated that the charge collection in a PSC with mesoporous TiO_2_ involves two separate electron transport pathways: one through the perovskite, and one through the mesoporous TiO_2_ (refs 45, 46). Following this, Heo *et al*. reported that surface deposition by sintering of bis(trifluoromethane)sulfonimide lithium salt (Li-TFSI) onto mesoporous TiO_2_ results in substantial improvements in the PCE and suppression of the hysteresis.

In this work, we show that mesoporous TiO_2_ can be n-doped in a facile and effective way by a similar lithium salt surface treatment. We show that the Li-doping enables faster electron transport within the mesoporous TiO_2_ electrodes and demonstrate that PSCs prepared on such electrodes achieve substantially higher performances compared with undoped electrodes, improving PCEs from 17 to 19.3% with negligible hysteresis behaviour (lower than 0.3%).

## Results

### X-ray photoelectron spectroscopy analysis

We applied the lithium ion surface treatment of the meso-porous TiO_2_ layer via spin coating of a LiTFSI solution (see the Methods section). After the deposition and solvent evaporation, the substrates were sintered at 450 °C for 30 min. The introduction of Li^+^ ions by the thermal diffusion modifies the surface of the particles. Together with the doping effect of the TiO_2_ an overlayer of LiO_2_ or LiOH could be formed^47^. Interestingly, also the formation of spinel structures like Li_4_Ti_5_O_12_ was observed with a synthetic procedure similar to the one employed here^48^.

**Figure 1:**
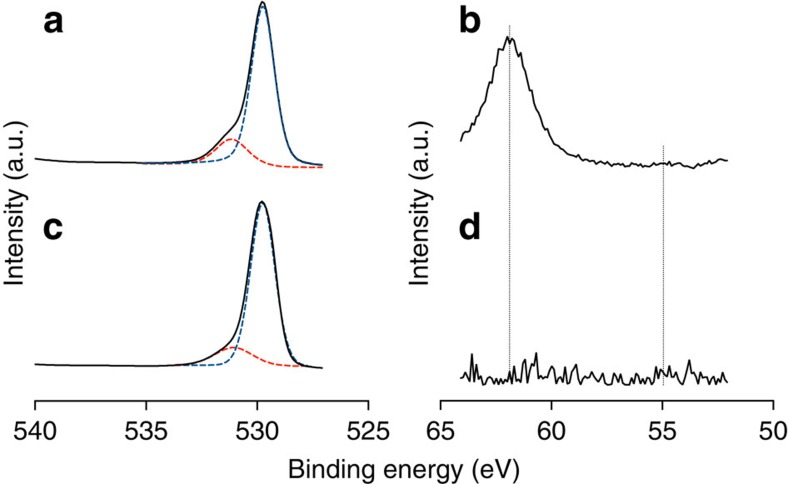
X-ray photoelectron spectroscopy. Doped and undoped mesoporous TiO_2_ layers for the O 1s peaks Li doped (**a**) and the undoped control (**c**), Ti 3*s* and Li 1*s* peaks slightly visible at 55 eV for Li-doped (**b**) and the signal for the undoped TiO_2_ here shown as reference, which reveals the absence of the peaks related to Ti 3*s* (dashed line at 61.5 eV) and Li 1*s* (**d**), dashed line at 54.9 eV.

We used X-ray photoelectron spectroscopy (XPS) to study the elemental composition of the Li-treated and untreated TiO_2_ after sintering. No traces of sulphur or fluorine from the LiTFSI precursor for the treated sample were detected and the high-resolution spectra for C 1*s* showed no difference between the Li-treated and untreated samples as seen in Supplementary Fig. 1. The O 1*s* spectra in Fig. 1a,c show that Li-treated TiO_2_ (Fig. 1a) has a more pronounced shoulder at the higher energy of the main peak compared with the untreated TiO_2_ (Fig. 1c). The deconvolution of this signal reveals a second small peak at 531.2 eV for the untreated sample and a much more pronounced one for the Li-treated one that has been previously assigned to the oxygen interaction with the lithium^47^. The Ti 2*p* spectra can be found in Supplementary Fig. 2, and no difference was detected for the treated and untreated samples. On the right hand side of Fig. 1, the spectrum from 50 to 65 eV for the Li-treated TiO_2_ (Fig. 1b) electrodes shows a peak corresponding to the Ti 3*s* and a weak, but distinct, signal for Li 1*s*. In the untreated TiO_2_ (Fig. 1d) no such signals, measured over a series of samples, could be detected in this energy region and it is here shown as noise. The Ti 3*s* peak at 61.5 eV reveals the presence of Ti^3+^ for Li-treated electrodes and thus indicates that the Li^+^ treatment induces a partial reduction of Ti^4+^ to Ti^3+^ within the TiO_2_ lattice^39^,^49^, which is not seen for the untreated electrode. Pathak *et al*. demonstrated that a small amount of species with valency +3 can passivate the electronic defects or trap states that originate from oxygen vacancies within the TiO_2_ lattice^37^. Accordingly, the Li^+^ doping mechanism is consistent with a passivation of electronic trap states resulting in improved charge transport properties and thus in better performing mesoporous TiO_2_ electrodes^50^.

### Charge extraction and electron transport analysis

To study the impact of the Li^+^ doping on the electronic states and the charge transport within the TiO_2_, we prepared solid-state DSSCs using Li^+^-doped mesoporous TiO_2_ as electron transporting layer. DSSCs were prepared according to the previously reported procedures^51^.

**Figure 2:**
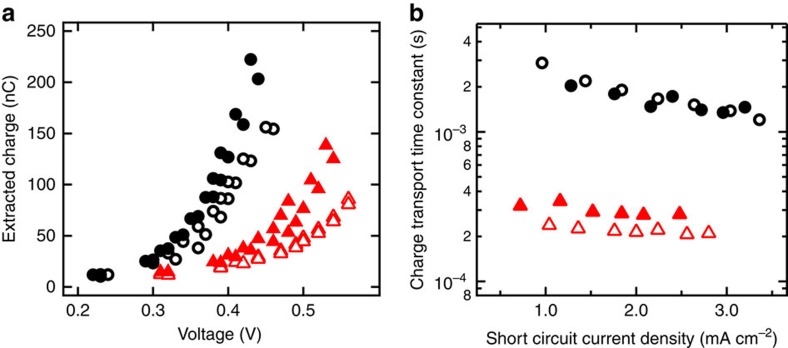
Charge extraction and electron transport time constant. (**a**) Charge extracted at open circuit as function of the voltage for no treated samples (black) and Li-doped samples (red) and (**b**) charge transport lifetime as function of the short circuit current density for solid-state dye-sensitized solar cells prepared without and with Li-doped mesoporous TiO_2_ (black and red markers, respectively). Two samples per condition are shown: open triangle, sample 1 Li-doped; closed triangle, sample 2 Li-doped; open circle, sample 1 no treated; closed circle, sample 2 no treated. For the charge extraction measurement (**a**), each sample was measured twice.

In the DSSC field, charge extraction is a well-established light-assisted technique, which qualitatively draws the density of state distribution below the TiO_2_ conduction band^52^. In Fig. 2a, we report the charge extracted from the DSSCs at open circuit condition as a function of the open circuit voltage. At the same open circuit voltage, the devices employing Li:TiO_2_ hold significantly less charges than the undoped TiO_2_ (for example at 0.46 V, Li:TiO_2_ and undoped TiO_2_ holds 44.3 and 154.3 nC, respectively). This suggests that the Li^+^ doping reduces the concentration of sub-bandgap states in the TiO_2_ supporting our previous conjecture that a partial reduction of Ti^4+^ to Ti^3+^ passivates the trapping states associated with oxygen vacancies within the TiO_2_ lattice.

From the same DSSCs, we extracted the charge transport time constant by intensity-modulated photocurrent spectroscopy^45^,^53^,^54^. The cell was biased at short circuit under light and the time constants were measured for different light intensities. In Fig. 2b, we compare the transport time constants for DSSCs prepared with Li^+^-treated and untreated TiO_2_ electrodes. Li^+^-treated devices display up to over one order of magnitude faster charge transport than the untreated devices over the whole range of current densities. To explain this trend, we note that charge transport in DSSCs is controlled by the electron transport in the TiO_2_ (ref. 41). In particular, it has been shown that the electronic transport in the TiO_2_ is limited by the temporary localization of electrons within sub-bandgap states, which can be passivated with different doping mechanisms^53^,^55^. Our results indicate that Li^+^ doping also reduces the concentration of sub-bandgap states (Fig. 2a) and improves the electronic transport within mesoporous TiO_2_ electrodes (Fig. 2b). As we observe the presence of Li^+^ ions within the TiO_2_ lattice (Fig. 1), we can regard this method as an effective way of n-doping via a facile post treatment of the mesoporous TiO_2_ films. Even though such doping strategies may be effective for improving DSSC performance, they may not necessarily benefit PSCs, as the latter already accomplish fast charge transport within the perovskite layer^17^. To further elucidate the effect of Li doping on the electron transport in mesoscopic perovskite solar cells, we performed light intensity-modulated photocurrent spectroscopy also on PSC^56^,^57^,^58^.

**Figure 3:**
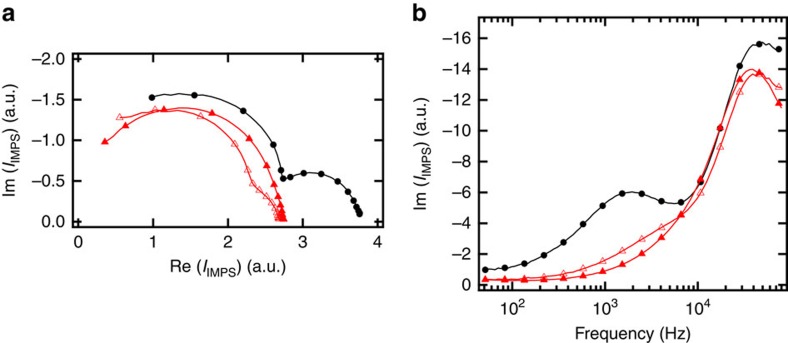
IMPS spectra. Nyquist plot (**a**) for PSCs prepared with no treated TiO_2_ electrode (in black at 0.15 mA cm^-2^) and with Li-doped TiO_2_ electrode at 0.07 mA cm^-2^ (open triangle) and 0.26 mA cm^-2^ (closed triangle). (**b**) Imaginary component of the same IMPS shown in **a** versus the frequency.

In Fig. 3a,b, we report the intensity modulated photocurrent spectroscopy (IMPS) spectra at different light intensities for PSCs prepared with mesoporous TiO_2_ films with and without the Li^+^-doping. The Nyquist plot of the untreated sample shows two distinct semicircles related to two different transport processes. In the treated sample, on the contrary, the slow time component (semicircle on the right) merges with the faster component (semicircle on the left) confirming that the TiO_2_ transport time is faster than that one observed in the control. These processes are resolved in frequency in Fig. 3b where the imaginary part of IMPS is plotted against the frequency. The electron transport time for a given pathway equals the inverse of the frequency of the corresponding peak^53^. In the case of the untreated TiO_2_ films, we observe two distinct peaks at 10^3^ and 10^4^–10^5^ Hz. Guillén *et al*. correlated these peaks with two separate electron transport pathways, running in parallel, involving the mesoporous TiO_2_ and the perovskite, respectively^45^. The peak at low frequency (10^3^ Hz) is directly linked to the electron transport in TiO_2_ and it shifts to higher frequency (faster transport) as the photocurrent density increases, as revealed also by the trends in Fig. 2b. By contrast, the high-frequency peak (10^4^–10^5^ Hz) is related to the charge transport through the perovskite and does not shift significantly with increasing the photocurrent density^45^. Therefore, at higher current density, the TiO_2_ and the perovskite peaks overlap at around 10^4^–10^5^ Hz. Interestingly, for the Li^+^-doped PSCs, the TiO_2_ peak at low frequency does not show up even for extremely low current density, such as 0.07 mA cm^−2^. As Li^+^-doping of TiO_2_ enables faster electron injection and transport in DSSCs, we infer from the lack of a low-frequency TiO_2_ peak for the Li^+^-treated PSCs (Fig. 3) that the electron transport through the TiO_2_ occurs at a similar rate as through the perovskite. This result also suggests that in PSCs employing doped TiO_2_ further improvements may originate from applying hole transporting materials with a higher hole mobility than the commonly used spiro-MeOTAD.

### PV characterization

**Figure 4:**
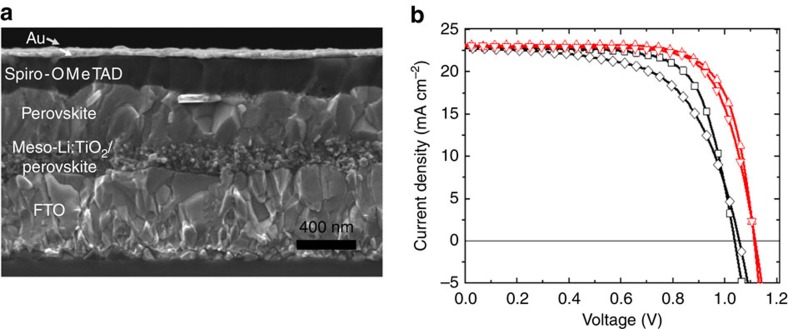
Morphology and *J*–*V* curves of the solar cells. (**a**) SEM cross-sectional image of the device. (**b**) Current density–voltage curves of the solar cells with and without the Li^+^ doping (red and black curve, respectively) collected under AM1.5 simulated sun light. Devices were masked with a black metal aperture of 0.16 cm^2^ to define the active area. The curves were recorded scanning at 0.01 V s^-1^ from forward bias to short circuit condition and *vice versa* with no device preconditioning such as light soaking or holding at forward voltage bias. Legend: black diamond, control forward scan; black square, control reverse scan; red down-pointing triangle, Li+-doped forward scan; red up-pointing triangle, Li+-doped reverse scan.

In Fig. 4a, we show the stack architecture used in this study composed of fluorinated tin oxide (FTO)/compact-TiO_2_/mesoporous-TiO_2_/perovskite/Spiro MeOTAD/gold. An X-ray diffraction pattern of the perovskite employed in solar cell fabrication is shown in Supplementary Fig. 3, where the peroskvite structure is detected. Figure 4b shows a typical current density–voltage (*J*–*V*) characteristic that we measured for PSCs with and without Li^+^-doped mesoporous TiO_2_. In Table 1, we summarize the device performance parameters, as extracted from the *J–V* curves in Fig. 4b, and the light intensity measured during each *J*–*V* scan. We note that the difference between the backward and the forward scans is significantly larger in the control than in the Li^+^-doped device. The short circuit currents are quite similar, whereas the Li^+^-doped device showed significantly larger open circuit voltage (about 60 mV) and 0.11 units higher fill factor. The current mismatch between the measured current density and the current density calculated by the incident photon-to-electron conversion efficiency (IPCE) over the Solar AM1.5 G spectrum was less than 2% (Supplementary Figs 4 and 5). The overall power conversion efficiency of devices employing the Li^+^-doped TiO_2_ electrodes was systematically higher than the devices employing the undoped scaffold (Supplementary Fig. 6). This result is consistent with the fact that the Li^+^-doping decreases the number of deep traps, which act as recombination centres and induces faster charge transport within the TiO_2_, improving the open circuit voltage and fill factor, respectively.

## Discussion

In summary, we demonstrated a doping mechanism that allowed preparing mesoporous TiO_2_ electrodes with superior electron properties. The doping can be accomplished with a facile post treatment of the mesoporous TiO_2_ making use of lithium salts to induce a partial reduction of Ti^4+^ to Ti^3+^ within the TiO_2_ lattice and passivating electronic defect states acting as nonradiative recombination centres. We exploit the Li^+^-doped mesoporous TiO_2_ electrodes to improve the maximum power conversion efficiency of perovskite solar cells from 17% to over 19%, which is comparable to the highest values reported in the literature.

## Methods

### Substrate preparation and Li-doping TiO_2_

Nippon Sheet Glass of 10 Ω sq^−1^ was cleaned by sonication in 2% Hellmanex water solution for 30 min. After rinsing with deionized water and ethanol, the substrates were further cleaned with ultraviolet ozone treatment for 15 min. Then, 30-nm TiO_2_ compact layer was deposited on FTO via spray pyrolysis at 450 °C from a precursor solution of titanium diisopropoxide bis(acetylacetonate) in anhydrous ethanol. After the spraying, the substrates were left at 450 °C for 45 min and left to cool down to room temperature. Then, mesoporous TiO_2_ layer was deposited by spin coating for 20 s at 4,000 r.p.m. with a ramp of 2,000 r.p.m. s^-1^, using 30 nm particle paste (Dyesol 30 NR-D) diluted in ethanol to achieve 150- to 200-nm-thick layer. After the spin coating, the substrates were immediately dried at 100 °C for 10 min and then sintered again at 450 °C for 30 min under dry air flow.

Li-doping of mesoporous TiO_2_ was accomplished by spin coating a 0.1 M solution of Li-TFSI in acetonitrile. The solution was prepared freshly before the application in nitrogen atmosphere. 150 μl were poured on 1.4 × 2.4 cm^2^ substrate. After 5 s of loading time, the spinning programme started with an acceleration of 1,000 r.p.m. s^−1^ to a final speed of 3,000 r.p.m., the substrate was left spinning for 30 s. Both Li^+^-doped and undoped electrodes were completed with a second calcination step at 450 °C for 30 min. After cooling down to 150 °C, the substrates were immediately transferred in a nitrogen atmosphere glove box for the deposition of the perovskite films.

### DSSC preparation procedure

Glass substrates for solid-state DSSCs were prepared following the same procedure used for PSC. 900 nm of mesoporous TiO_2_ (Dyesol 30 nrd ethanol diluited ) were deposited by spin coating at 4,000 r.p.m. After sintering, the substrates were cooled down to 70 °C and immersed in 0.1 mM solution of dye (Y123) in 1:1 mixture of acetonitrile and *tert*-butyl alcohol for 30 min. After the dyed films were rinsed in abundant acetonitrile, the hole conductor was applied by spincoating at 2,000 r.p.m. for 20 s. The hole transport composition and the following steps to complete the DSSCs were identical to what used for PSCs.

### Perovskite precursor solution and film preparation

The perovskite films were deposited from a precursor solution containing FAI (1 M), PbI_2_ (1.1 M), MABr (0.2 M) and PbBr_2_ (0.2 M) in anhydrous dimethylformamide/ dimethylsulphoxide (4:1 (v:v)) solution. The perovskite solution was spin coated in a two-step programme at 1,000 and 4,000 r.p.m. for 10 and 30 s, respectively. During the second step, 100 μl of clorobenzene was poured on the spinning substrate 15 s prior the end of the programme. The substrates were then annealed at 100 °C for 1 h in nitrogen-filled glove box.

We note that the perovskite precursor solution for this paper was prepared with a different composition from what reported by Jeon *et al*., who used an equimolar amount of FAI and PbI_2_ to achieve a certified PCE of 17.9% with the mixed halide and cation formulation, (FAPbI_3_)_0.85_(MAPbBr_3_)_0.15_ (ref. 24). Interestingly, we observed a systematic improvement in PCE moving away from the equimolar concentration for FAI and PbI_2_ towards 10 mol% lower stoichiometric amount of FAI.

### Hole transporting layer and top electrode

After the perovskite annealing, the substrates were cooled down for few minutes and a spirofluorene-linked methoxy triphenylamines (spiro-OMeTAD, from Merck) solution (70 mM in chlorobenzene) was spun at 4,000 r.p.m. for 20 s. The spiro-OMeTAD was doped with bis(trifluoromethylsulfonyl)imide lithium salt (Li-TFSI, from Aldrich), tris(2-(1H-pyrazol-1-yl)-4-*tert*-butylpyridine)- cobalt(III) tris(bis(trifluoromethylsulfonyl)imide) (FK209, from Dyenamo) and 4-*tert*-Butylpyridine (TBP, from Aldrich)^40^,^41^,^59^. The molar ratio of additives for spiro-OMeTAD was: 0.5, 0.03 and 3.3 for Li-TFSI, FK209 and TBP, respectively. Finally, 70–80 nm of gold top electrode was thermally evaporated under high vacuum.

### PV device testing

The solar cells were measured using a 450-W xenon light source (Oriel). The spectral mismatch between AM1.5G and the simulated illumination was reduced by the use of a Schott K113 Tempax filter (Präzisions Glas & Optik GmbH). The light intensity was calibrated with a Si photodiode equipped with an IR-cutoff filter (KG3, Schott) and it was recorded during each measurement. Current–voltage characteristics of the cells were obtained by applying an external voltage bias while measuring the current response with a digital source meter (Keithley 2400). The voltage scan rate was 10 mV s^-1^ and no device preconditioning was applied before starting the measurement, such as light soaking or forward voltage bias applied for long time. The starting voltage was determined as the potential at which the cells furnishes 1 mA in forward bias, no equilibration time was used. The cells were masked with a black metal mask (0.16 cm^2^) to estimate the active area and reduce the influence of the scattered light. The devices were characterized 2 days after their preparation.

### Charge extraction technique

Charge extraction measurement were performed with Autolab potentiostat PGSTAT30 driven by NOVA software. The procedure for the charge extraction comprised four steps. First, the cell was kept for 10 s in dark at short circuit. At this stage, the carriers eventually accumulated in the intrinsic capacitances of the device were discharged. Then, the potential was brought to open circuit and the light was switched on for an equilibration time of 10 s. The light was then switched off and the open circuit voltage decay was monitored for a defined decay time *T*_d_. In the last step, the cell was brought back to short circuit condition from *V*_TD_ (voltage at time *T*_d_) and the discharge current was measured. The integration over the time (starting from *T*_d_) of this current gave the value of charge stored at the voltage *V*_TD_.

## Additional information

**How to cite this article:** Giordano, F. *et al*. Enhanced electronic properties in mesoporous TiO_2_ via lithium doping for high-efficiency perovskite solar cells. *Nat. Commun.* 7:10379 doi: 10.1038/ncomms10379 (2016).

## Supplementary Material

Supplementary Information Supplementary Figures 1-6

## Figures and Tables

**Table 1:** Solar cell performance parameters.

	**Scan direction**	***J***_**sc**_**(mA cm**^**−2**^**)**	***V***_**oc**_**(V)**	**FF**	**PCE (%)**	**Light intensity (mW cm**^**−2**^**)**
Li^+^	Backward	23.0	1.114	0.74	19.3	98.1
doped	Forward	23.1	1.118	0.72	19.0	
Control	Backward	22.7	1.038	0.72	17.1	99.4
	Forward	22.7	1.056	0.61	14.7	

FF, fill factor; PCE, power conversion efficiency.

Short circuit photocurrent (*J*_sc_), open circuit voltage (*V*_oc_), FF extracted from the data in Fig. 4.
