# Supplementary material for: Enhanced electronic properties in mesoporous TiO2 via lithium doping for high-efficiency perovskite solar cells
Source: Nat Commun. 2016 Jan 13;7:10379. doi: 10.1038/ncomms10379 (PMC4735553; doi:10.1038/ncomms10379)
Supplement: Supplementary Information — Supplementary Figures 1-6 [file ncomms10379-s1.pdf]

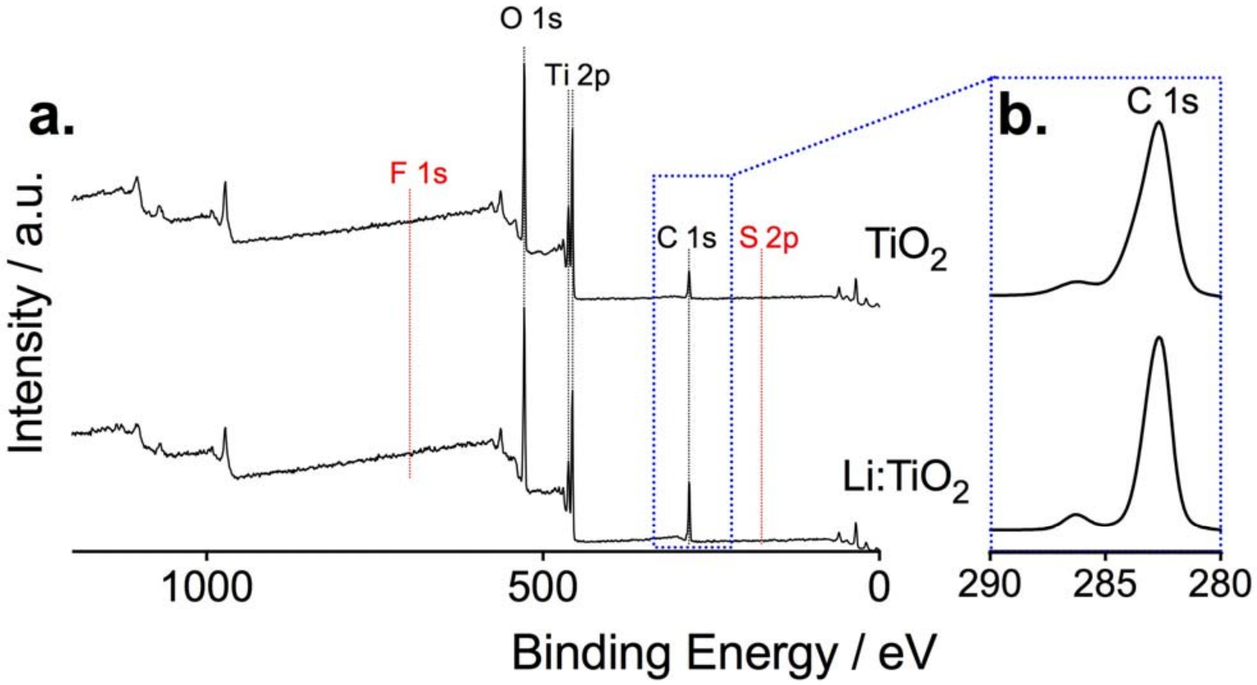

Supplementary Figure 1. a) XPS survey spectra of Li-doped and undoped TiO_2 electrodes showing Ti 2p, O 1s and C 1s peaks. The lines in red depict the position of elements that are not present in the XPS spectrum but that are present in the precursor solution of LiTFSI. b) high resolution spectra for C 1s for doped and undoped TiO_2.

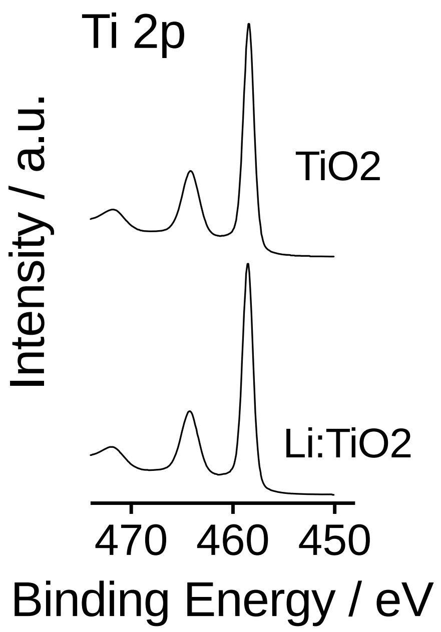

Supplementary Figure 2. XPS Ti 2p spectra of Li-doped and undoped TiO_2 electrodes.

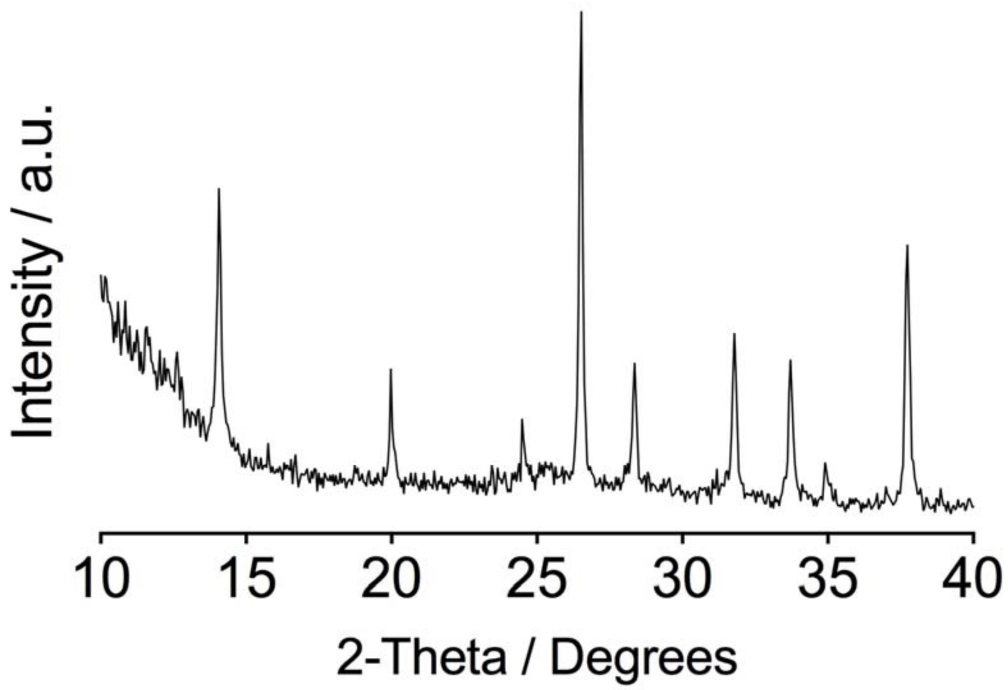

Supplementary Figure 3. XRD pattern of the $(FAPbI_3)_{0.85}(MAPbBr_3)_{0.15}$ perovskite material used in this study.

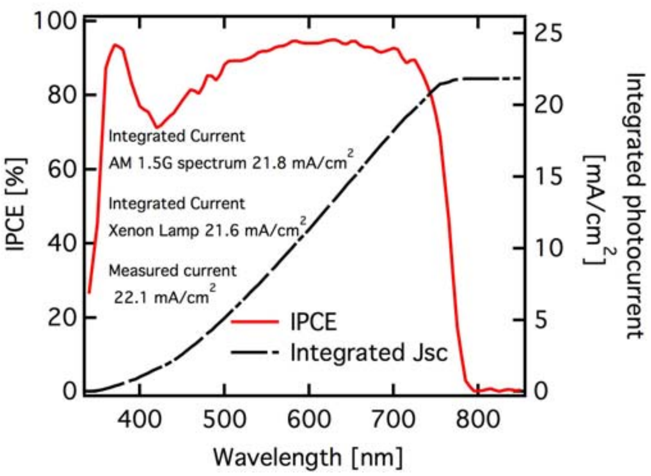

Supplementary Figure 4. IPCE measurement of a device employing Li doped scaffold. Incident photon-to-current conversion efficiency measurements were carried from the monochromated visible photons, from Gemini-180 double monochromator Jobin Yvon Ltd. (UK), powered by a 300 W xenon light source (ILC Technology, USA) superimposed on a 1mW/cm^2 LED light. The monochromatic incident light was passed through a chopper running at 8 Hz frequency and the on/off ratio was measured by an operational amplifier.

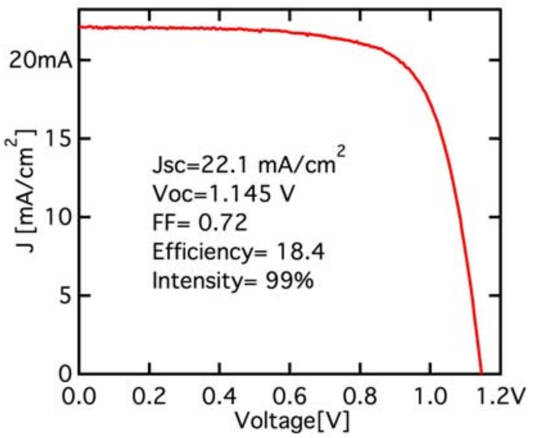

Supplementary Figure 5. IV measurement and PV values of the device used for the IPCE measurement

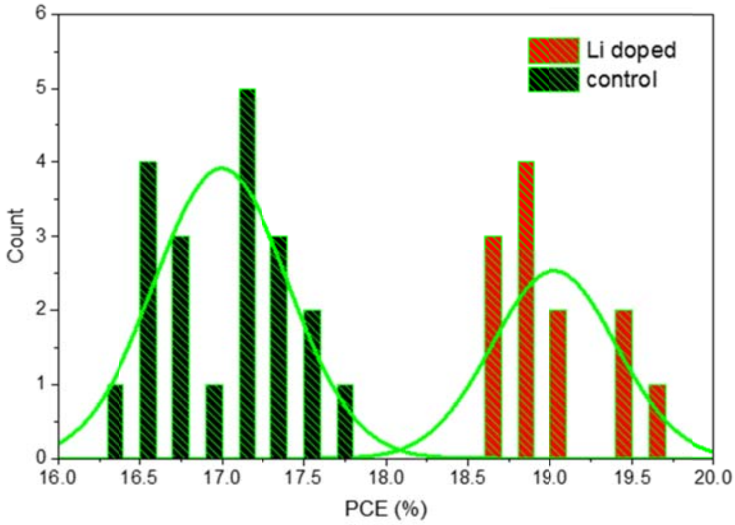

Supplementary Figure 6. Histogram with normal distribution curve of PCE.
